# Hippocampal Functional Connectivity Mediates the Impact of Acceptance on Posttraumatic Stress Symptom Severity

**DOI:** 10.3389/fpsyt.2020.00753

**Published:** 2020-07-30

**Authors:** Wi Hoon Jung, Nam Hee Kim

**Affiliations:** ^1^ Department of Psychology, Daegu University, Gyeongsan, South Korea; ^2^ Maumtodac Psychiatric Clinic, Ansan, South Korea; ^3^ Suwon Smile Center for Criminal Victims, Suwon, South Korea

**Keywords:** acceptance, functional connectivity, posttraumatic stress symptoms, resting-state fMRI, neural marker

## Abstract

Investigation in posttraumatic stress disorder (PTSD) shows a negative association between patients’ degrees of acceptance (the willingness to face unwanted private experiences while pursuing one’s values and goals) and those of clinical symptom severity, suggesting that experiential acceptance is a protective factor of symptoms or an early indicator of resilience after trauma. However, neural mechanisms involved in the relationship between these two variables have yet to be elucidated. Thus, we here investigate whether there are neural mechanisms mediating such relationship using whole-brain voxel-level mediation analysis with seed-based resting-state functional connectivity (RSFC) maps generated by hippocampal subregion seeds in accident survivors (n = 33). We found that the correlation between patients’ acceptance and symptom severity was mediated by the RSFC strength between left hippocampal body and left lateral occipital cortex adjacent to superior parietal cortex, the areas related to flashbacks. Our result provides novel evidence that hippocampal RSFC mediates the effect of experiential acceptance on posttraumatic stress symptom severity. If further refined and validated, the finding may aid to the identification of biomarkers to intervention and prevention programs for patients with PTSD.

## Introduction

Posttraumatic stress disorder (PTSD) is a debilitating condition that develops after encountering traumatic events, such as direct exposure to life-threatening accidents, sexual or physical assaults, domestic violence, or indirect witnessing others experience traumatic events. More than half of adults have experienced at least one traumatic event during their lifetime, and approximately 7% of them will develop PTSD (1–3). Given the high prevalence of the disorder, the detection of reliable early indicators of vulnerability and the development of early interventions have received increasing interest in recent years and have contributed the understanding of the pathophysiological mechanisms of PTSD. In this regard, many researchers have used neuroimaging features to determine vulnerability- and disease-specific neural markers in an attempt to forecast the onset or course of the disorder.

The investigation demonstrates that posttraumatic stress symptoms (PTSS) are developed and maintained by habitual efforts to avoid trauma-related thoughts, emotions, and memories, suggesting that they may be decreased by acceptance as psychological flexibility (4–6). Indeed, investigation shows that while experiential avoidance, defined as an unwillingness to experience unwanted internal events, is associated with greater PTSS severity, experiential acceptance is associated with grater psychological adjustment after exposure to traumatic events, (7). It is thus suggested that lower levels of experiential acceptance may be one of the individual difference factors associated with vulnerability to PTSD (7, 8).

Neuroimaging techniques have been employed to identify neural mechanisms underlying PTSD across a variety of patients with different traumatic events (9–12), yet exact neural mechanisms involved in the pathophysiology of PTSD are still a subject of debate. Most neuroimaging studies to date have pointed to a potentially critical role for the areas associated with emotion and memory processing in PTSD, including medial prefrontal cortex, anterior cingulate cortex, insula, precuneus, amygdala, and hippocampus (9, 10, 12). Of these, alterations in the hippocampus, the region that plays a role in memory and contextual processing, are highly reproducible across structural and functional magnetic resonance imaging (10, 11). For instance, a large body of literature over the last two decades has demonstrated smaller hippocampal volumes (10, 13) and altered hippocampal activation [including hypo- (14, 15) or hyper-responsiveness (16)] during a variety of tasks, including memory, fear learning, and extinction.

Recently, resting-state functional connectivity (RSFC) approaches have been used as invaluable research tools for investigating neural mechanisms underlying cognitive ability and clinical in humans (17–19). In patients with PTSD, seed-based RSFC analyses exhibited aberrant functional connectivities between each of different seed regions (including hippocampus, amygdala, anterior cingulate cortex, and anterior insula) and remote brain areas (20–22). For example, reduced hippocampal RSFC to the posterior cingulate cortex or amygdala has been observed in patients with PTSD compared to controls [(23–25); though see (22)]. However, despite of the different functional and structural connectivities depending on hippocampal subregions (26, 27), previous studies with regard to the hippocampus have mainly investigated the region as a unitary structure. Therefore, examining hippocampal RSFC in a subregion level may further advance our understanding of the neurobiology of PTSD.

Taken together, previous studies have revealed multiple brain regions associated with the PTSS severity, with particular emphasis on the importance of hippocampus, and showed a negative relationship between PTSS severity and acceptance. However, to date there are no published reports providing a brain mediator of the relationship between these two variables. Here, we therefore investigated whether there are neuronal mechanisms mediating such relationship. Building on previous findings, we hypothesized that the acceptance predicts the PTSS severity *via* the RSFC to the hippocampus, particularly its specific subregion. To test this hypothesis, we used a whole-brain voxel-level mediation analysis with the seed-based RSFC map to each of hippocampal subregions, (i.e., the head, body, and tail) as a potential brain mediator, after confirming a significant correlation between acceptance and PTSS severity.

## Materials and Methods

### Participants

We recruited 95 participants who experienced one of the various types of accidents as traumatic experiences, aged 18–65 years, and visited the trauma center at a university hospital between March 2016 and May 2019. Specifically, accidents include car accidents, falling accidents, or workplace accidents. Life Events Checklist of the Diagnostic and Statistical Manual of Mental Disorder-Fifth Edition (28) was utilized to define trauma. Their psychiatric diagnosis was made using the Structured Clinical Interview for DSM-IV Axis I Disorders-Clinician Version. No all participants had any of the following clinical conditions: primary psychotic diagnoses such as schizophrenia, schizoaffective disorder, and schizophreniform disorder, serious medical condition sufficient to interfere with their participation in this study, intellectual disabilities, or neuro-cognitive impairment. Many of the initial sample (n = 60) refused to revisit the hospital to take part in MRI scans of the present study. The remaining 35 who agreed to take part in MRI scans were enrolled in this imaging study. After preprocessing the imaging data, two participants out of 35 were excluded due to excessive head motions. Therefore, 33 were included in the final analysis. We confirmed that the final sample size was rational to obtain meaningful results, based on a power analysis using online application Monte Carlo Power Analysis for Indirect Effects of mediation models [(29) https://schoemanna.shinyapps.io/mc_power_med/]. The analysis resulted in power of 0.75 for the sample size of 33, when assuming the correlations of 0.65 for paths a and b and of 0.60 for path c’ as well as standard deviations of 1 for all these paths as input model values (see *Mediation Analysis* below). This study was approved by the Institutional Review Board of an affiliated university hospital (AJIRB-MED-MDB-15-120). All participants received a comprehensive description of the study and then signed an informed consent form.

### Acceptance and Clinical Symptom Measures

All participants were assessed with the Acceptance and Action Questionnaire-II (AAQ-II) to quantify the degree of acceptance (6). This scale measures psychological acceptance or flexibility to experience unwanted private experiences (30). To evaluate the severity of clinical symptoms, patients were assessed with the Clinician-Administered PTSD Scale-5 (CAPS-5) (31), Beck Depression Inventory-II (BDI-II) (32), and Dissociative Experiences Scale-II (DES-II) (33) to measure the severity of PTSS, subjective depressive symptoms, and subjective dissociative symptoms, respectively.

### Image Acquisition and Preprocessing

All brain images were acquired with a 3T Philips Achieva scanner. All participants were asked to abstain from smoking, caffeine and other stimulants from midnight the night before his/her scan. Resting-state functional magnetic resonance imaging (RS-fMRI) data collection was performed using a fast field echo (FFE)-echo planar imaging (EPI) sequence (repetition time/echo time = 2000/30 ms, flip angle = 80°, filed of view =240 mm, voxel size = 3 × 3 × 4.5 mm^3^, 31 axial slices, 200 volumes, total scan time = 6 min 40 s). During RS-fMRI scanning, participants were instructed to keep their eyes closed and to not think about anything. After scanning, a simple questionnaire was administered to confirm they had not fallen asleep. A high-resolution 3D T1-weighted scan was also collected using a FFE sequence (TR/TE = 9.8/4.6 ms, FA = 8°, FOV = 240 mm, voxel size = 1 × 1 × 1 mm^3^, 160 sagittal slices) for anatomical reference.

Image preprocessing was performed using SPM12 (http://www.fil.ion.ucl.ac.uk/spm) and DPARSFA toolbox (http://rfmri.org/DPARSF/) (34). After discarding the first four volumes for each subject, images were corrected for slice timing and head motion. To reduce motion-related artifacts, the data were checked with the following criterion for excessive head motion; (i) six motion parameters > 1.5 mm or 1.5° in any direction and (ii) a mean frame-wise displacement (FD) > 0.5 mm (>2 SD from the group mean) (35). Two participants were excluded according to the criterion. The remaining data were co-registered to the T1 structural image of each individual subject and then the coregistered images were segmented into gray matter, white matter, and cerebrospinal fluid (CSF). Next, the images were further regressed out of the following nuisance variables: Friston’s 24 motion parameters (i.e., 6 motion parameters, 6 motion parameters one time point before, and the 12 corresponding squared items), head motion scrubbing regressors (FD > 0.5, one volume before, and two volumes after the bad time point), five principal components estimated from both white matter and CSF regions using a component-based noise correction (CompCor) method (36), and a linear trending term. The CompCor method estimates the amount of noise from physiological and other spurious sources by deriving principal components from noise masks without external monitoring of physiological fluctuations (36). Additionally, it can better account for voxel specific phase differences in physiological noise, compared to the average signal from WM and CSF regions (37). The residual images were normalized to the Montreal Neurological Institute (MNI) space and were then smoothed with a 4 mm full-width half-maximum Gaussian kernel. Finally, time series were band-pass filtered (0.01–0.1 Hz) to reduce the effect of low frequency drift and high frequency physiological noise.

### Seed-Based Functional Connectivity Analysis

**Figure 1:**
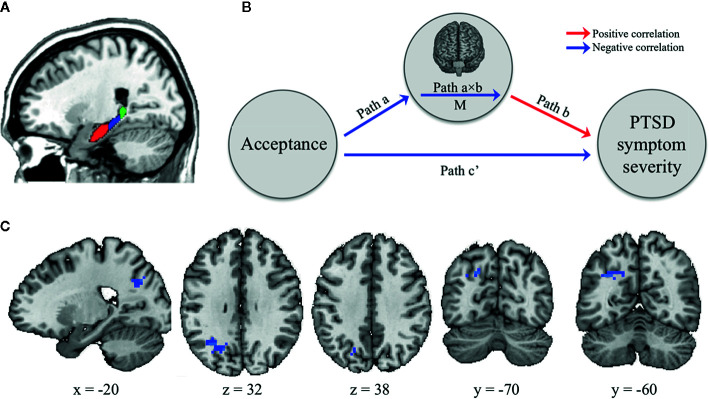
The brain mediator of acceptance on posttraumatic stress symptom (PTSS) severity. **(A)** The boundaries for the head (red), body (blue), and tail (green) of the hippocampus as seed regions **(B)** Whole-brain search model for brain mediators. Whole-brain voxel-wise mediation was used to determine the region significantly functionally connected with hippocampal subregions on their resting-state functional connectivity (RSFC) maps as a brain mediator in the relationship between acceptance and PTSS severity. Path a indicates that the acceptance predicts the RSFC strength between hippocampal seed and specific brain areas. Path b indicates this RSFC strength predicts PTSS severity, controlling for acceptance. Path a×b as a mediator shows that the hippocampal-specific brain area RSFC explains a significant proportion of the covariation between acceptance and PTSS severity. Path c’ indicates the relationship between these two variables controlling for M. Notable is that the colors of arrows are determined based on results after completing the analysis. The red and blue arrows represent positive and negative relationships, respectively. **(C)** Brain mediators of the relationship between the acceptance and PTSS severity. The RSFC strength between left hippocampus body seed and left lateral occipital cortex adjacent to superior parietal cortex (LOC/SPC) mediated the impact of acceptance on PTSS severity.

To create seed-based RSFC maps to each of the subregions of the hippocampus (i.e., head, body, and tail) for each hemisphere, we first identified left and right hippocampus from the Harvard–Oxford atlas and then the subregions were manually defined in a manner similar to that described by previous studies (38, 39) (**Figure 1A**). The head–body boundary was identified in the sagittal plane, while the body–tail boundary was identified in the coronal plane as the first slice where the fimbria of the fornix was evident. For each subject, Pearson correlation coefficients as functional connectivity strengths were calculated between the mean time series for each hippocampal seed and time series for all other voxels throughout the rest of the brain. These correlation coefficients were converted into z-values using Fisher r-to-z value transformation.

### Mediation Analysis

Mediation analysis tests whether the direct effect of an independent variable (X) on a dependent variable (Y) can be explained by the indirect influence of a mediator variable (M). Using the Mediation Toolbox (https://github.com/canlab/MediationToolbox), we investigated whether the effect of the acceptance (X) on the PTSS severity (Y) was explained indirectly by the RSFC strength (M), particularly the seed-to-voxel functional connectivity to each of hippocampal subregions (**Figure 1B**). Participants’ age, sex, BDI-II, and DES-II scores were included as covariates of no interest in the model. The path model jointly tested the following three effects of interest in every single voxel (10,000 bootstrapped samples per voxel): (i) the effect of the acceptance (X) on the RSFC (M) (indirect effect, path a); (ii) the effect of the RSFC (M) on the PTSS severity (Y) by controlling the effect for the acceptance (X) (indirect effect, path b); and (iii) the mediation effect (a × b) which is defined as the reduction of the relationship between the acceptance (X) and PTSS severity (Y) (total relationship, path c) by including the RSFC (M) into the model (direct path, path c’). All results were thresholded at p < 0.05 family-wise error rate corrected based on cluster extent, with primary threshold of p < 0.001, two-tailed and uncorrected.

## Results

### Descriptive Data Analysis

**Table 1** shows demographic and clinical characteristics of participants. All participants were right-handedness and 14 subjects (42%) were female. The mean [SD] of age was 40.36 [11.10] years old. The mean [SD] of AAQ-II, CAPS-5, BDI-II, and DES-II as indices of acceptance, PTSS severity, depressive symptom severity, and dissociative symptom severity was 39.88 [11.03], 25.27 [10.99], 25.15 [13.62], and 17.08 [23.89], respectively.

**Table 1:** Demographic and clinical characteristics of participants.

Variables	Mean ± SD or n (%)
**Demographic**	
Age (years)	40.36 ± 11.10
Sex (male/female)	19 (57.6)/14 (42.4)
Education	
≤ High school	18 (54.6)
≥ College/university	15 (45.4)
**Clinical**	
Trauma type	
Traffic accident	29 (84.9)
Falling/injuries	5 (15.1)
AAQ-II	39.89 ± 11.03
CAPS-5 severity	25.27 ± 11.0
BDI-II	25.15 ± 13.62
DES-II	17.08 ± 23.89
Diagnosis	
PTSD	21 (63.6)
Acute stress disorder	3 (9.1)
Adjustment disorder	9 (27.3)
Time since trauma (months)	6.57 ± 7.54
<1	9 (27.3)
1–3	9 (27.3)
3–12	8 (24.2)
≥12	7 (21.2)

SD, standard deviation; AAQ-II, Acceptance and Action Questionnaire-II; CAPS-5, Clinician-Administered PTSD scale-5; BDI-II, Beck Depression Inventory-II; DES-II, Dissociative Experiences Scale-II; PTSD, posttraumatic stress disorder.

The acceptance was negatively correlated with PTSS severity (r = −0.81, p < 0.001). This correlation remained significant after controlling for age, sex, BDI-II and DES-II scores (r = −0.58, p < 0.001).

### Mediation Effects of Functional Connectivity

While the RSFC maps to the left and right hippocampal head and tail seed regions exhibited no mediation effects between the acceptance and PTSS severity, those to the left hippocampal body seed (but not the right hippocampal body seed) showed a significant mediation effect (at p < 0.05, cluster-level correction, with voxel-level p < 0.001); especially, one significant cluster was found in left lateral occipital cortex adjacent to superior parietal cortex (LOC/SPC; Brodmann area [BA] 19/7; MNI x, y, z = −24, −63, 33; p-value < 0.001; **Figure 1C**). In other words, the acceptance was negatively correlated with the RSFC from the left hippocampal body seed to the left LOC/SPC (path a). This RSFC was positively correlated with PTSS severity (path b). Finally, the map of the path a × b effect showed a negative mediation in the left LOC/SPC, resulting from the acceptance-associated reductions in RSFC (negative path a) and a positive RSFC–PTSS severity relationship (positive path b).

## Discussion

This is the first investigation to examine whether there are neural mechanisms mediating the impact of the acceptance on PTSS severity. Especially, we used seed-based RSFC maps to each of hippocampal subregions as a potential brain mediator. Consistent with previous results (7), we first found a significantly inverse correlation between the acceptance and PTSS even controlling for age, sex, BDI-II and DES-II scores. The result from our mediation analysis revealed that the RSFC strength between the left hippocampal body and left LOC/SPC mediates the impact of acceptance on PTSS severity.

Previous studies have demonstrated the involvement of the hippocampus and parahippocampal gyrus (PHG) in the development of PTSD (11). For instance, a reduced hippocampal volume in patients with PTSD, compared to controls, is consistently observed across meta-analysis (13) and multisite MRI studies (10). Albeit inconsistent across findings from functional neuroimaging studies, aberrant hippocampal activity is also repeatedly reported, showing decreased (14, 15) or increased activity (16) in the region. Moreover, PTSS severity was associated with altered parahippocampal activity (40, 41) and aberrant hippocampal RSFC (42).

The hippocampus is involved in declarative memory, such as episodic and autobiographical memory, and fear conditioning, and the PHG is functionally closely related to the hippocampus (i.e., learning and emotion) (27, 43). The hippocampus is also thought to contribute to the process of integrating context into emotion memories (44). Like the hippocampus, visual areas are associated with memory processing. A recent neural decoding study showed that visual areas can retain visual information held in working memory, over delay period without the presentation of physical stimulus (45). However, based on literatures, while the hippocampus is more engaged in memory consolidation and retrieval (though it is also involved in memory encoding), the visual areas may be more related to memory encoding (46). Intriguingly, a recent study with normal volunteers has found that visual imagery vividness is positively associated with PHG activity and negatively with visual area activity (47). Taken together, considering the involvement of these regions found here in memory processing and their different roles in visual imagery, dysfunctional connectivity between the hippocampal complex and visual area in patients may give rise to vivid images of the trauma they experienced (called “flashback”, which is a defining feature of PTSD).

Clinically, acceptance involves decentering from immediate, habitual reactivity, observing moment-to-moment transitions in thoughts, sensations, and emotions (48). LOC is involved in object perception (49) and SPC serves functions related primarily to somatosensory system, or tactile perception (50). These areas are strongly connected to hippocampus and this hippocampal-occipitoparietal network involves the elaboration of autobiographical memory retrieval (51, 52). Taken together, all of these previous findings support our aforementioned interpretation of the negative correlation between acceptance and the RSFC strength between the hippocampal body and LOC/SPC, suggesting that with higher acceptance, decreased connectivity in the sensorimotor and visual area during elaboration of autobiographical memory retrieval could reduce the vividness of traumatic memories. It could be helpful to process the traumatic memory within the window of tolerance in patients with psychological trauma and contribute to lower levels of PTSS, such as flashbacks. This idea is supported by results from previous studies to investigate neural signatures of flashbacks in PTSD; flashbacks in patients with PTSD was associated with increased activation in sensorimotor and visual areas and decreased activation in the parahippocampal gyrus, midbrain, and precuneus (53). PTSD patients’ flashback intensity correlated with rCBF in the left hippocampal area (54).

A recent study has proposed the Hippocampal Encoding/Retrieval network (HERNET) Model and provided neural evidence of the model, showing that anterior portions are associated with encoding processes from the external/dorsal attention network, while posterior portions are associated with retrieval processes from the internal attentional network (55). A previous seed-based RSFC in human showed different functional and structural connectivity patterns depending on a given seed area out of hippocampal subregions (i.e., the head, body, and tail of left hippocampus); the hippocampal head seed was correlated with the limbic areas, the regions associated with affective processing; the hippocampal body seed was linked to the cognitive and affective regions including the limbic areas as well as perceptual processing regions including lateral temporal and occipital cortices; and the hippocampal tail seed was correlated with frontal and temporal areas (56, 57). Considering different connectivity patterns and roles of hippocampal subregions, our finding may reflect deficits of retrieval processes in PTSD.

In the current study, the mediation effect of hippocampal RSFC between the acceptance and PTSS severity was observed only in left hemisphere. Though we did not have hypotheses about hemispheric lateralization, much previous work has suggested potential hemispheric specialization of the hippocampus. The rodent studies have argued left-right dissociation of hippocampal memory processes, showing that left CA3 input produced more long-term potentiation at CA1 synapses than right CA3 input (58) and only left CA3 was associated with performance on an associative spatial long-term memory task (59). The functional lateralization of the human hippocampus is less well studied, though two recent studies have reported reduced hippocampal volume in patients with PTSD compared to controls, particularly and the left hippocampal volume was more reduced than the right side (10, 13).

The current study has several limitations to mention. First, the study is limited by the moderate sample size and power, though we recruited a large number of subjects (n = 95) in our effort to study patients with traumatic accidents. As in this study, previous studies have consistently reported a strong negative correlation between the acceptance and PTSS severity (60, 61), and moreover, found that PTSS severity is associated with left hippocampal RSFC even in smaller patient samples (n > 25) than ours (42). For these reasons, we believe that our findings reported here is valuable, though a replication in a larger sample size is needed. Second, subjective depressive and dissociative symptoms of some participants were quite high, which could have confounding effects. To reduce the effects, BDI and DES scores have been included in the model as covariates. Third, the level of education was quite different among participants. However, an exploratory analysis revealed no significant differences in the levels of acceptance and PTSS between two group (t-/p-values = 1.49/0.15 for acceptance; t-/p-values = −1.81/0.08 for PTSS severity) divided by the education level [the high school group (n = 18) versus the college/university group (n = 15)]. Finally, the present study included only accident survivors who visited specialized university hospital center for trauma, and they had been exposed to quite severe trauma. A comparison between other psychological trauma survivors and other clinical populations would be valuable to determine whether the observed relationship is specific to individuals with severe psychological trauma or not. Therefore, future studies comparing with other clinical populations in larger sample sizes are needed to expand our understanding of how the acceptance and the RSFC interact with trauma-related processes and their influence on the PTSS.

In summary, using whole-brain voxel-level mediation analysis, the current study revealed the mediating role of the RSFC between the left hippocampus and left LOC/SPC in the relationship between acceptance and PTSS severity in patients, though further research with a larger sample is needed to confirm this novel finding. Based on previous findings showing the effect of acceptance-based therapy on PTSS (7, 62), our results may aid to the identification of biomarkers to intervention and prevention programs for patients with PTSD.

## Data Availability Statement

All datasets generated for this study are included in the article/supplementary material.

## Ethics Statement

The studies involving human participants were reviewed and approved by the Institutional Review Board of Ajou University Hospital. The patients/participants provided their written informed consent to participate in this study.

## Author Contributions

NK designed the research. WJ analyzed the data. All authors contributed to the article and approved the submitted version.

## Funding

This study was supported by a grant from the Korean Mental Health Technology R&D Project, Ministry of Health & Welfare, Republic of Korea (HM15C1058) and was supported by the National Research Foundation of Korea (NRF) grant funded by the Korea government (MSIT) (NRF-2019R1G1A1098972).

## Conflict of Interest

The authors declare that the research was conducted in the absence of any commercial or financial relationships that could be construed as a potential conflict of interest.
